# III-Nitrides empower miniaturized spectral imager in ultraviolet

**DOI:** 10.1038/s41377-025-02132-1

**Published:** 2026-01-23

**Authors:** Yuji Zhao, Tao Li, Boon Ooi

**Affiliations:** 1 Department of Electrical and Computer Engineering, Rice University, Houston, TX 77005 USA; 2 Photonics Laboratory, Computer, Electrical, and Mathematical Sciences and Engineering Division (CEMSE), King Abdullah University of Science and Technology (KAUST), Thuwal, 23955-6900 Saudi Arabia; 3 Department of Electrical, Computer, and Systems Engineering, Rensselaer Polytechnic Institute, Troy, NY USA

**Keywords:** Spectrophotometry, Optical sensors

## Abstract

A high-performance miniaturized on-chip spectral imager operating in the ultraviolet region is demonstrated based on an AlGaN/GaN cascaded photodiode array. This work extends spectral imaging into the ultraviolet regimes by leveraging the mature III-nitride technologies and establishes a scalable pathway toward massive production of compact, high-resolution spectral imagers.

## Abstract (web summary)

A miniaturized on-chip spectral imager based on III-nitride cascaded photodiodes achieves 0.62 nm accuracy and sub-10 ns response, extending spectral imaging into the ultraviolet regime.

## Graphical abstract

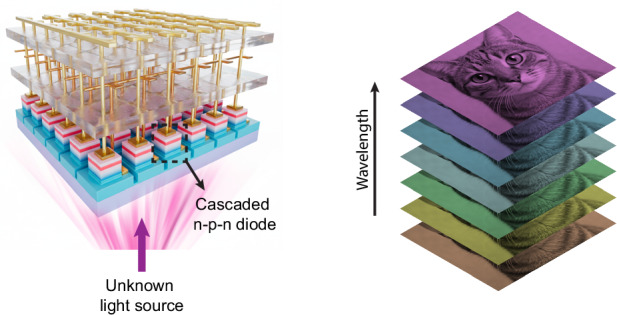

III-nitride semiconductors have been successfully employed as a key material for fabricating highly efficient optoelectronics such as light-emitting (LED, laser) and detection (photodiode) devices^1^. Thanks to their direct and tunable bandgaps, which cover a wide spectrum range from deep ultraviolet to near infrared, III-nitride optoelectronics are mainly developed for applications, including outdoor/indoor lighting, display, and optical communication. However, they have not tapped into the development of spectrometers/spectral imagers, which are powerful optical instruments capable of simultaneously recording spatial and spectral information, thereby providing comprehensive, high-dimensional insight into an observed object. This technology has found extensive applications and produced far-reaching impacts across diverse fields, including spectral analysis, remote photo-sensing, and biomedicine^2,3^.

Conventional spectral imaging instruments typically consist of bulky optical components and mechanical scanning units for both spectral and spatial dimensions. While these systems offer high accuracy and resolution, their large footprint and heavy weight significantly limit portability and deployment versatility.

Driven by the growing demand for compact yet accurate and high-resolution spectral imaging, numerous innovative mechanisms and system architectures have been proposed to miniaturize imagers operating in the visible and infrared spectrum^4–8^. However, in the ultraviolet (UV) and deep-ultraviolet (DUV) ranges—crucial for applications such as biopharmaceutical analysis, organic compound characterization, and molecular detection—miniaturized spectroscopic imaging technologies remain scarce. This is primarily due to the intrinsic challenges associated with material limitations, fabrication constraints, and structural complexity.

To overcome these obstacles, an international research team led by Prof. Haiding Sun at the University of Science and Technology of China has demonstrated a miniaturized on-chip spectrometer and spectral imager operating in the UV and DUV regions, based on an AlGaN/GaN cascaded-diode array^9^. The device structure is illustrated in Fig. 1a. Each spectral imager comprises a two-dimensional array of identical cascaded n-p-n diodes, with each cascaded diode consisting of two asymmetric vertical p-n junctions. The operating principle, summarized in Fig. 1d, relies on tuning the voltage applied to the cascaded n-p-n diode to adjust its wavelength-dependent responsivity.

**Fig. 1:**
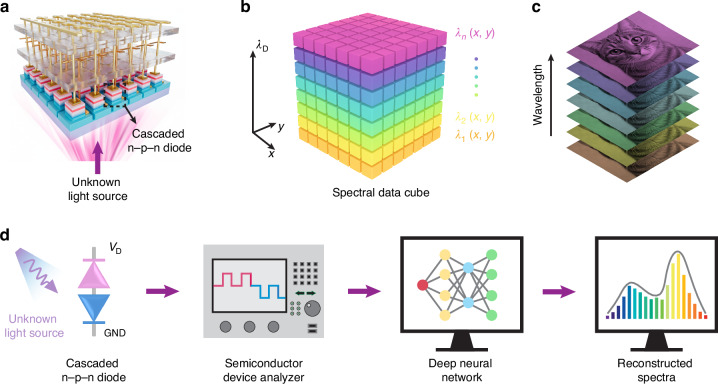
Illustration of the structure and working principle of III-nitride cascaded photodiode-based miniaturized on-chip spectral imager. **a** A schematic illustration of a fabricated miniaturized spectral imager chip. **b** A 2D array of cascaded n-p-n diodes (pixels) within the spectral imager reconstructs a three-dimensional spectral data cube. **c** Schematic of application examples using a spectral imager to analyze the spectral information of images. **d** Working principle of a cascaded photodiode for building a miniaturized on-chip spectral imager

Specifically, when illuminated by an input light with an unknown but fixed spectrum, sweeping the applied voltage shifts the diode’s peak responsivity across different wavelength regions, thereby encoding the spectral information into the voltage-dependent output current. By feeding this voltage–current relationship into a pre-trained deep neural network (DNN), the spectrum—i.e., the wavelength-optical-power distribution—of the input light can be accurately reconstructed. Utilizing a 2D array of such tunable diodes enables simultaneous acquisition of spatial and spectral information, reconstructing a three-dimensional spectral data cube of the imaging plane, as illustrated in Fig. 1b, c.

This study introduces two significant breakthroughs in the field of spectral imaging. First, it extends the frontier of miniaturized spectral imaging technologies into the ultraviolet (UV) and even deep-ultraviolet (DUV) regimes for the first time. The fabricated spectral imager exhibits outstanding performance, achieving a peak wavelength accuracy of 0.62 nm and a sub-10 ns temporal response across the 260–365 nm spectral range.

More importantly, the concept and framework presented in this work offer remarkable scalability and extensibility by leveraging the relatively mature III-nitride technology. The on-chip spectral imagers are wafer-scale fabricated from GaN/AlGaN thin films epitaxially grown on sapphire substrates. Because the fabrication process is fully compatible with existing advanced semiconductor mass-production technologies, the device feature size can be further reduced to the sub-micron or even nanometer scale, enabling higher-resolution on-chip spectroscopic imaging while potentially lowering the cost of conventional spectroscopic imagers by orders of magnitude.

Furthermore, by tuning the composition and doping characteristics of the compound semiconductor materials—either using III-nitride alloys or employing other II–VI (e.g., CdS, ZnO) or III–V (e.g., GaAs, InP) materials which act as light-absorptive layers—the operational bandwidth of this miniaturized spectral imaging architecture can be easily expanded from the UV into the visible and even infrared regions.

In summary, this study demonstrates a high-performance, miniaturized spectrometer and spectral imager operating in the UV and DUV regimes, enabled by a combination of tunable cascaded AlGaN/GaN photodiode arrays and advanced DNN-based spectral reconstruction algorithms within a single on-chip platform. The demonstrated framework provides a promising and scalable pathway toward the mass production of compact, portable, and high-resolution spectral imaging devices, opening new avenues for future on-chip spectroscopic applications.
